# Transcatheter bidirectional Glenn shunt guided by real-time MRI

**DOI:** 10.1186/1532-429X-17-S1-O23

**Published:** 2015-02-03

**Authors:** Kanishka Ratnayaka, Toby Rogers, William Schenke, Jonathan R Mazal, Marcus Y Chen, Merdim Sonmez, Michael Hansen, Ozgur Kocaturk, Anthony Z Faranesh, Robert J Lederman

**Affiliations:** 1 NIH/NHLBI, Bethesda, MD, USA

## Background

Children with single ventricle physiology require multiple open heart surgeries for palliation, including sternotomies and cardiopulmonary bypass. The reduced morbidity of a catheter-based approach is attractive. We hypothesize real-time multiplanar MRI guidance enables closed-chest percutaneous bidirectional Glenn shunt because of arbitrary-plane imaging capability.

## Methods

Ten swine underwent transcatheter bidirectional Glenn procedures under MRI at 1.5T. An MRI antenna-needle was advanced from the superior vena cava (SVC) into the target pulmonary artery (PA) bifurcation using real-time MRI guidance. A caval-pulmonary sheath introduced endografts. Balloon-expansion secured a proximal end-to-end caval anastomosis that also occluded the azygos, and a distal end-to-side pulmonary anastomosis that preserved blood flow to both branch pulmonary arteries.

## Results

**Figure 1:**
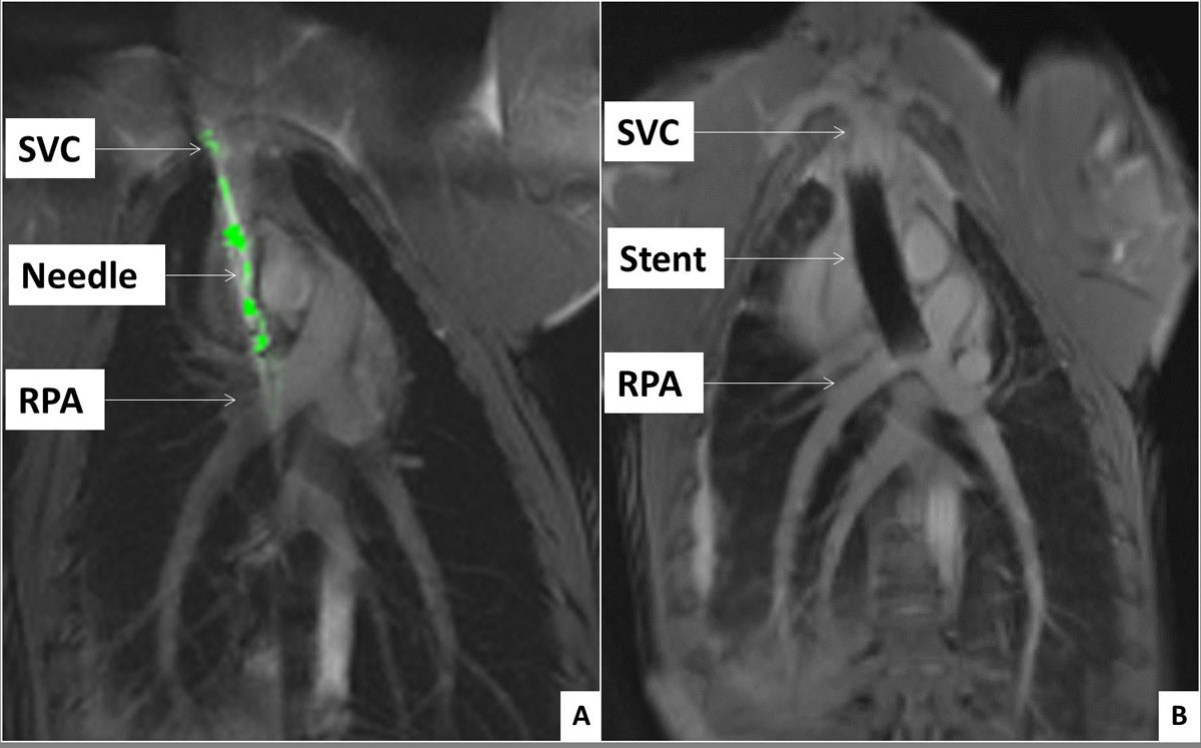
A depicts real-time MRI needle access of adjacent vessels [superior vena cava (SVC) and right pulmonary artery (RPA)]. Figure 1B shows final bidirectional Glenn shunt with anastomosis of SVC and RPA using endograft.

Real-time MRI needle access of adjacent vessels (SVC and RPA; figure 1A), endograft delivery, and superior vena cava conduit to pulmonary arteries (figure 1B) was successful in all animals (n = 10; weight = 23.6 ± 3.3 kg). All survived the procedure without complications. Post-procedural MRI and X-ray angiography showed patent Glenn shunts with bidirectional pulmonary artery blood flow. This new animal model was characterized by sudden conversion to bidirectional Glenn physiology. Six survived to one week follow up.

## Conclusions

MRI guidance enabled a complex closed-chest beating heart pediatric transcatheter structural heart procedure that otherwise requires open surgery and cardiopulmonary bypass. In this study, MRI alone guided a wholly percutaneous Glenn shunt that preserved bidirectional pulmonary artery flow. Clinical translation would require minor refinement of catheter tools.

## Funding

Z01-HL005062 (NHLBI/NIH DIVISION OF INTRAMURAL RESEARCH).

